# The COVID-19 Pandemic Led to a Small Increase in Changed Mentality Regarding Infection Risk without Any Change in Willingness to Be Vaccinated in Chronic Diseases Patients

**DOI:** 10.3390/jcm10173967

**Published:** 2021-09-01

**Authors:** Cyril Breuker, Anne Marie Guedj, Mathilde Allan, Loick Coinus, Nicolas Molinari, Nicolas Chapet, François Roubille, Moglie Le Quintrec, Véronique Duhalde, Julien Jouglen, Philippe Cestac, Jean Marie Kinowski, Stéphanie Faure, Marie Faucanie, Laura Lohan, Maxime Villiet, Romain Altwegg, Ariane Sultan

**Affiliations:** 1 Department of Clinical Pharmacy, CHU Montpellier, University of Montpellier, 34000 Montpellier, France; m-allan@chu-montpellier.fr (M.A.); l-coinus@chu-montpellier.fr (L.C.); n-chapet@chu-montpellier.fr (N.C.); l-lohan_descamps@chu-montpellier.fr (L.L.); m-villiet@chu-montpellier.fr (M.V.); 2 PhyMedExp, CHU Montpellier, INSERM, CNRS, University of Montpellier, 34295 Montpellier, France; a-sultan@chu-montpellier.fr; 3 Department of Endocrinology, University Hospital of Nîmes, 30900 Montpellier, France; anne.marie.guedj@chu-nimes.fr; 4 Department of Statistics, CHU Montpellier, University of Montpellier, 34000 Montpellier, France; n-molinari@chu-montpellier.fr; 5 Department of Cardiology, PhyMedExp, CHU Montpellier, INSERM, CNRS, University of Montpellier, 34295 Montpellier, France; f-roubille@chu-montpellier.fr; 6 Department of Nephrology, CHU Montpellier, University of Montpellier, 34000 Montpellier, France; m-lequintrec-donnette@chu-montpellier.Fr; 7 Department of Pharmacy, Toulouse University Hospital, 31300 Toulouse, France; duhalde.v@chu-toulouse.fr (V.D.); jouglen.j@chu-toulouse.fr (J.J.); cestac.p@chu-toulouse.fr (P.C.); 8 Centre for Epidemiology and Population Health Research (CERPOP), UMR 1027, INSERM, University of Toulouse (UPS), 31000 Toulouse, France; 9 Department of Pharmacy, CHU Nîmes, 30000 Nîmes, France; jean.marie.kinowski@chu-nimes.fr; 10 Department of Gastroenterology, CHU Montpellier, University of Montpellier, 34000 Montpellier, France; s-faure@chu-montpellier.fr (S.F.); r-altwegg@chu-montpellier.fr (R.A.); 11 Clinical Research and Epidemiology Unit, CHU Montpellier, University of Montpellier, 34000 Montpellier, France; m-faucanie@chu-montpellier.fr; 12 Department of Endocrinology-Diabetology-Nutrition, CHU Montpellier, University of Montpellier, 34000 Montpellier, France

**Keywords:** people living with diabetes, vaccination coverage, COVID-19 pandemic, infection risk, chronic disease

## Abstract

The objective of this study was to assess the impact of the COVID-19 pandemic on patients’ perceptions regarding infection risk and vaccination in subjects suffering from chronic diseases. A prospective observational multicentric study conducted from December 2020 to April 2021 in three French University Hospitals. Patients with chronic diseases were proposed to complete a questionnaire regarding the impact of the COVID-19 pandemic on infectious risk knowledge and vaccination. A total of 1151 patients were included and analyzed (62% of which were people with diabetes). The COVID-19 pandemic increased awareness of infectious risks by 19.3%, significantly more in people with diabetes (23.2%, from 54.4% to 67.0%, *p* < 0.01) when compared to the other high-risk patients (12.5%, from 50.5% to 56.8%, *p* = 0.06). Respectively, 30.6% and 16.5% of patients not up-to-date for pneumococcal and flu vaccines reported wanting to update their vaccination due to the COVID-19 pandemic. By contrast, the proportion of patients against vaccines increased during the COVID-19 pandemic (6.0% vs. 9.5%, *p* < 0.01). The COVID-19 pandemic has led to a small increase in awareness regarding the risks of infection in patients with chronic diseases, including people with diabetes, but without any change in willingness to be vaccinated. This underlines the urgent need to sensibilize people with diabetes to infection risk and the importance of vaccination.

## 1. Introduction

Influenza and pneumococcal diseases are common infectious diseases with higher morbi-mortality in subjects living with chronic diseases, including diabetes. Thus, in people with diabetes, it is well demonstrated that both flu and pneumoniae are more severe, associated with higher risk of hospitalization, and mortality [1]. These infections could be prevented by vaccination in accordance with the recommendations published by the health authorities and the learned societies [2,3]. Vaccination coverage rates in these target populations remain low and below national health objectives. According to the ministry of health, in France for the winter of 2019, in populations at risk of severe forms of the disease, influenza vaccination coverage was less than 50%, whereas it should be 75% [4]. In people with diabetes, it is around 68% [5]. On March 11 2020, WHO declared the SARS-CoV-2 to be a pandemic. All European countries implemented physical distancing measures as an emergency response to contain COVID-19 and its associated death toll, especially in high-risk patients [6]. Several studies reported that COVID-19 is more severe in subjects living with chronic diseases, notably obesity and type 2 diabetes mellitus, with a higher need for intensive care unit and increased death risk [7]. According to the WHO COVID-19 Dashboard of the 19 May 2021, there are more than 160 million cases of COVID-19 and more than 3.3 million deaths. However, despite COVID-19 potential gravity, Lin and colleagues showed that COVID-19 vaccine hesitancy is increasing worldwide in a systematic review of 126 surveys on COVID-19 vaccination intentions published between 1 January and 20 October 2020 [8]. This vaccine hesitancy did lead to a low vaccine coverage for influenza, pneumococcal, and COVID-19, all infections that represent a risk of severe infections, hospitalization, and death. Larson et al. in 2016 found the French population to be the most skeptical about vaccination, around 45% [9]. Around COVID-19, fake news had circulated extensively since the beginning of the pandemic, thus making the general audience doubt the veracity of health and political authorities concerning information around the SARS-CoV-2 [10]. The World Health Organization’s Director-General declared the global ‘over-abundance’ of COVID-19 information an ‘infodemic’ [11]. Thus, the current COVID-19 pandemic may have contributed to a change in patients’ perceptions regarding both the higher risk of infection in some subjects and the potential benefit of vaccination. In this context, we believe that it is important to evaluate the infection risk perception, coverage, and feeling about vaccination during the pandemic of COVID-19 in different at-risk populations. We hypothesized that (1) there is a lack of knowledge regarding infectious risk and vaccination coverage of recommended vaccines among patients at risk of infection; and (2) the COVID-19 pandemic has changed the knowledge and perceptions of at-risk patients regarding their infectious risk and the potential value of vaccination, which could offer an opportunity to improve the coverage in a next step.

Therefore, the primary objective of this present study was to assess the impact of the COVID-19 pandemic on patients’ perceptions regarding infection risk in an at-risk population, including subjects living with diabetes and subjects suffering from other chronic diseases. The secondary objectives were to assess the vaccination coverage of mandatory and recommended vaccines and the impact of the COVID-19 pandemic on vaccination perception.

## 2. Materials and Methods

### 2.1. Study Design, Setting and Participants

From December 2020 to April 2021, we conducted a prospective multicentric observational study in three French University Hospitals (University Hospital of Montpellier, Nîmes, and Toulouse). Participation was proposed to all adult patients at risk of infection, according to French authorities, hospitalized (full-time hospitalization or day hospitalization) or consulting in five medical units (one immuno-rheumatology unit of University Hospital of Toulouse; one endocrinology unit of University Hospital of Nîmes; one endocrinology unit, one cardiology unit, and one hepato-gastroenterology unit of University Hospital of Montpellier) [12]. Inclusion criteria were to be over 18 years of age and to have at least one chronic condition or comorbidity with an increased risk of infection. Chronic conditions or comorbidities with an increased risk of infection were considered as cardiovascular history (cardiomyopathy, heart failure, coronary artery disease, congenital heart disease), diabetes, chronic renal failure, primary or acquired immune deficiencies (immunosuppressive therapy or treatment with anti-TNF-alpha), obesity (body mass index > 30 kg/m^2^).

A questionnaire was specifically developed for this study and contained specific questions regarding (i) the perception of infectious risk and the impact of the COVID-19 pandemic on this perception, (ii) knowledge of recommended vaccines (influenza and pneumococcal vaccines, and, if >65 years old, herpes zoster vaccine), (iii) knowledge on the vaccinations performed (influenza and pneumococcal vaccines, and, if >65 years old, herpes zoster vaccine) and the impact of the COVID-19 pandemic on these vaccinations, (iv) the traceability of these vaccinations, (v) the reasons for not performing the mandatory and recommended vaccinations, and (vi) feelings about vaccines (favorable, unfavorable, mixed, or no opinion) and the impact of the COVID-19 pandemic.

### 2.2. Intervention

The paper questionnaire was anonymous and completed by the patient themself during their hospitalization for inpatients or before the medical consultation for outpatients. The completed questionnaires were centralized at the Montpellier University Hospital and the data were entered into a database. An online questionnaire realized with the Sphinx software was used for the patients followed in teleconsultation.

### 2.3. Data and Statistical Analysis

Self-reported data were provided by the questionnaire, including demographic (age, sex), clinical (chronical disease and duration), immunization coverage (Tdap, influenza, and pneumococcal vaccines, and, if >65 years old, herpes zoster vaccine), and feeling about vaccines. Patient characteristics and questionnaire responses were expressed as frequency and proportion for categorical variables, and mean ± standard deviation (SD) for continuous variables. Comparisons were made with Student’s *t*-test or the Mann–Whitney U-test for continuous variables, and with chi-square or Fisher’s exact test for categorical variables, depending on the distribution of variables and conditions for applying the tests.

### 2.4. Ethics Approval

All patients who accepted to complete the form were prospectively included. All procedures performed in this study involving human participants were in accordance with the 1964 Helsinki declaration and its later amendments. Oral consent was obtained before inclusion in the study. The study protocol was approved by the Institutional Review Board of our university hospital (Comité Local d’Ethique Recherche, IRB-MTP_2020_12_202000673) and was registered on ClinicalTrials.gov (Impact of the COVID-19 Pandemic on Vaccination, COVIDVacImpac study NCT03422484).

## 3. Results

### 3.1. Patient Characteristics

During the study period, 1439 patients suffering from various chronic diseases were included; 288 were removed from the analysis due to missing data. The population is described in Table 1, the mean age was 54.9 ± 16.7 years, 52.2% were men, and the mean duration of primary disease was 15.5 ± 12.2 years. Most patients were included in medical consultations (55.6%). The subpopulation of people with diabetes was composed of 714 patients (62.0%), subjects were more often men (57.7 ± 16.0 vs. 50.4 ± 17.0, when compared to patients without diabetes, *p* < 0.01), older (57.7 ± 16.0 vs. 50.4 ± 17.0 years, *p* < 0.01), and had a longer history of chronic disease (17.4 ± 12.9 vs. 12.2 ± 10.2 years, *p* < 0.01).

### 3.2. Patient Feelings about Infectious Risk

In total, 52.9% of patients feel that their medical condition is associated with an increased risk of infection (Table 2). The COVID-19 pandemic significantly increases this rate to 63.1% (*p* < 0.01), with a significantly higher increase in people with diabetes (increase of 23.2%, from 54.4% to 67.0%, *p* < 0.01) compared to other high-risk subjects (increase of 12.5%, from 50.5% to 56.8%, *p* = 0.06). More specifically, 62.2% of subjects felt vulnerable for flu infection, with a higher percentage in people with diabetes compared to subjects suffering from other chronic diseases (65.1% vs. 57.4%, *p* < 0.01). However, only 32.3% of patient felt vulnerable for pneumococcal infection, without any difference regarding diabetes status.

### 3.3. Knowledge about Vaccine and Immunization Coverage

In total, 186 (17%) subjects could cite influenza as one of the recommended vaccines, with a lower proportion in people with diabetes (15.4% vs. 19.4%, *p* = 0.09). However, 59.7% of subjects declared to be vaccinated against flu, with a significantly higher proportion in people with diabetes (64.6% vs. 52.0%, *p* < 0.01). Regarding vaccination against pneumococcal infection, 62 (5.7%) patients could cite pneumococcal as a recommended vaccine, with a lower percentage in people with diabetes (3.4% vs. 9.2%, *p* < 0.001). In total, 28.4% of patients report to be vaccinated against pneumococcus, with, again, a lower proportion in people with diabetes (24.8% vs. 33.6%, *p* < 0.01).

The main reasons for not being vaccinated against flu are fear of potential adverse effects (24.1%), the lack of usefulness as patients do not consider themselves at risk of infection (22.1%), and lack of vaccine trust (17.7%) (Table 3). People without diabetes were more likely to be unaware that the flu vaccine is recommended for their condition (15.0% vs. 5.1%, *p* < 0.01). Conversely, more people with diabetes think the flu vaccine is ineffective (18.1% vs. 8.0%, *p* < 0.01), whereas, for the non-vaccination against pneumococcus, the main cause is the lack of knowledge of the recommendation of the vaccination (73.6%). The COVID-19 pandemic has an impact on the willingness to be vaccinated, with, respectively, 30.6% and 16.5% of patients whose pneumococcal and influenza vaccines are not up-to-date that want to be vaccinated, with a significantly higher proportion of people without diabetes compared to people with diabetes (20.6% vs. 12.8%, *p* < 0.03).

### 3.4. Feelings about Vaccines and the Impact of the COVID-19 Pandemic

In subjects that reported not to be “for” vaccines (against, mixed, or no opinion, *n* = 487), the COVID-19 pandemic positively changed for 35.3% of them their opinion on vaccines, without any difference regarding diabetes status (37.2% vs. 32.4%, *p* = 0.28). This change was explained by the potential benefits of vaccination (41.3%), the access to more information on vaccination (30.5%), and the fear of being hospitalized (34.7%) or dying (16.8%). However, the percentage of anti-vax subjects significantly increases during the COVID-19 pandemic (6.0% vs. 9.5%, *p* < 0.01) (Table 4).

## 4. Discussion

Our study demonstrates that the COVID-19 pandemic significantly increased patients’ awareness regarding infectious risk associated with their condition (from 52.9% to 63.1%; i.e., +19.3%). This increase was even greater in people with diabetes (54.4% to 67.0%; i.e., +23.2%) compared to subjects suffering from other chronic disease (50.5% vs. 56.8%; i.e., +12.5%). However, despite this increase in infectious risk awareness linked to the COVID-19 pandemic, we observe, paradoxically, a decrease in confidence in vaccines. In addition, although the COVID-19 pandemic has been evolving for one year, our results demonstrate that awareness about infectious risks and vaccination coverage are still insufficient in chronic disease patients. 

### 4.1. Infectious Risks, Immunization Recommendations, and Chronic Diseases

Our results are particularly important given the observation of insufficient vaccination coverage, as already mentioned, and low awareness of infectious risks in a high-risk population. It is well known that subjects with chronic diseases and comorbidities, such as diabetes, are at increased risk of infection, including influenza and pneumonia, and, more recently, COVID-19 [13]. COVID-19 is not only a pulmonary infection, but also leads to extra pulmonary symptoms with cardiovascular diseases, severe acute renal failure, and digestive, hepatic, and cerebral diseases [14]. Many studies have investigated for risk factors for severe COVID-19 disease. Thus, patients who were male, with advanced age, obesity, hypertension, diabetes, malignancy, coronary heart disease, chronic liver disease, chronic obstructive pulmonary disease, or chronic kidney disease were more likely to develop severe COVID-19 symptoms, or were also associated with high mortality [15,16].

In our study, we evaluated the impact of the COVID-19 pandemic on the perception of infectious risk in a population at risk of severe illness, including outpatients and inpatients. We particularly studied the perception of subjects living with diabetes (62.0% of the population), as epidemiological studies have quickly and consistently pointed out diabetes as one of the major comorbidities associated with COVID-19 and affecting its severity [17]. Already, in April 2020, patients with diabetes have been listed as people at higher risk for severe illness from COVID-19 by several health authorities and learned medical societies [7].

In our study, we observe that the COVID-19 pandemic has led to an increase in awareness of the infectious risk, with the increase significantly higher in people with diabetes. However, we also observe that this increase is quite low at one year after the appearance of COVID-19, and despite the significant media coverage of the pandemic. Thus, 36.9% of the population at risk of severe infection are not aware of infectious risks. This underlines the importance of therapeutic education, particularly on the risk of infection in this at-risk population. This lack of education on infectious risk is also reflected in the knowledge of recommended vaccines for at-risk patients, with only 17.0% and 5.7% of patients citing influenza and pneumococcus, respectively, and vaccination coverage, with 59.7% and 28.4% of patients reporting being vaccinated against influenza and pneumococcus, respectively. One of the main reasons for not being vaccinated is the absence of feeling at risk and the lack of knowledge of the vaccination recommendation for pneumococcus, emphasizing the need for education for these patients. We find an effect on awareness of the infectious risk in connection with the COVID-19 pandemic, with 30.6% and 16.5% of patients not up-to-date with the influenza and pneumococcal vaccines wishing to be vaccinated. This increase in influenza vaccination coverage is also found in the general French population, with an increase of 8% (47.8% in 2019/2020 to 55.8% in 2020/2021) [18].

### 4.2. Information on Infectious Risk and Benefits for Patients

Our study began one year after the outbreak of the new coronavirus disease (COVID-19) was first reported in Wuhan City, China, in December 2019, and after the second lockdown in France [19]. The discovery of this new coronavirus and the announcement by the World Health Organization that COVID-19 was a global pandemic had an impact on public awareness and behavior, particularly on the search for information on COVID-19 [20,21]. Thus, media have played a crucial role in communication regarding risk, information about the virus, contamination, and treatment, as well as protective behavior and applicable rules. Thus, S. Li et al. highlighted that COVID-19-related information online and risk awareness are key factors associated with US residents’ engagement in various preventive behaviors [22]. However, studies on risk awareness have mainly focused on general populations and have analyzed sociodemographic variables, such as education level or gender [23]. However, few studies focused on subjects suffering from chronic diseases, such as people with diabetes [24,25,26]. Flint et al. explored the awareness and attitudes of an at risk of severe COVID-19 adult population in the UK during the lockdown period [26]. A large proportion of participants reported that they were ‘very concerned’ about infection, spread, and potential impact of COVID-19, which demonstrates that informing subjects can change their feelings and behavior. Moreover, participants living with diabetes were more likely to practice social distancing (OR 2.44, 95% CI 1.25 to 4.90) and to wear protective apparel (OR 2.17, 95% CI 1.13 to 4.14). This study is comparable to ours in terms of sample size (1026 and 1151 patients), mean age of subjects (54.6 and 54.9 years), and proportion of subjects living with diabetes (52.4% and 62.0%). In their study, Yan et al. showed that the 585 participants with diabetes perceived themselves to be at a higher risk and were more worried about being infected with COVID-19 when compared to individuals without diabetes (*p* < 0.001) [27]. However, the population of this study is much younger than ours (89% of the population is less than 50 years old), and the chronic pathologies of patients without diabetes are not known. Regarding the benefits for patients, several studies showed a decrease in community-acquired [28] and nosocomial infections [29,30] during the COVID 19 pandemic compared to previous years. These decreases in virus circulation and infection transmission in both outpatient and inpatient settings are related to the direct benefit of the implementation of distancing, prevention, and isolation measures.

### 4.3. COVID-19 and Vaccines

In our study, however, despite this increase in awareness of the infectious risks associated with the COVID-19 pandemic and the willingness of some patients to be vaccinated against influenza or pneumococcus, there was a significant slight decrease in patients’ feelings about vaccination during the pandemic, with 53.7% and 50.3% of patients for vaccination after and during the COVID-19 pandemic, respectively. This decrease and the low rate of patients in favor of vaccination can be explained by several factors. The first one is that vaccine skepticism in France is well described, with the French population being one of the most skeptical about vaccination [9]. The second one may be a lack of or insufficient physicians’ attention regarding vaccine recommendations during medical consultations due to time constraints and, sometimes, to hesitancy or relative ignorance [5,31,32]. Misinformation about health-related subjects represents a public health threat. During the COVID-19 pandemic, fake news has circulated extensively since the beginning of the pandemic, thus making the general audience doubt the veracity of health and political authorities concerning information around COVID-19 and vaccination [33]. Moreover, misinformation and misbeliefs can influence willingness to follow the recommendations by health and political authorities on vaccination. A number of studies found that fake news remains the main cause of vaccine hesitancy [34,35,36].

Some limitations of our study should be noted. First, it provides a cross-sectional analysis at a given time and we are not able to describe changes in opinion over time, but our study was carried out almost a year after the onset of COVID-19 disease, which may limit this bias. Second, we did not study the impact of COVID-19 on attitudes, lifestyles, and actions. Finally, our population included only a few patients over 75 years of age, and only includes patients with chronic disease managed by hospital-based specialist physicians. Despite these limitations, the key strengths of the study include: (i) our recruitment method, as this study was offered to all patients present during the study period, avoiding the biases associated with self-recruitment methods, such as motivated sample selection, that can be found in studies using online survey methodology; (ii) representativeness of our population, with different chronic diseases, participation of three university hospitals, including six medical departments, and medical departments from different specialties; and (iii) our sample size that allows comparison between subjects.

## 5. Conclusions

In conclusion, in our population of subjects with advanced chronic diseases and long duration of illness, there is still a lack of awareness of subjects with chronic diseases regarding infectious risk and vaccine recommendation. Further, the COVID-19 pandemic has led to a small increase in awareness of the risks of infection in patients with chronic diseases, including people with diabetes, without any change in willingness to be vaccinated. This underlines the urgent need to sensibilize people with diabetes to infection risk and the importance of vaccination. 

## Figures and Tables

**Table 1:** Characteristics of the population.

	Person without Diabetes	Person with Diabetes	*p*
*n*	*n* = 437	*n* = 714	
Sex, male (*n*, %)	199 (45.5)	402 (56.3)	<0.01
Age (years), mean (SD) (*n* = 1157)	50.4 (±17.0)	57.7 (±16.0)	<0.01
Center			<0.01
Montpellier	288 (65.9)	485 (67.9)	
Nîmes	120 (27.5)	226 (31.6)	
Toulouse	29 (6.6)	3 (0.4)	
Medical unit			<0.01
Endocrinology–Diabetology–Nutrition Department, Montpellier	51 (11.7)	464 (65.0)	
Hepato-Gastroenterology Department, Montpellier	174 (39.8)	6 (0.8)	
Cardiology and Vascular Diseases Department, Montpellier	30 (6.9)	4 (0.6)	
Nephrology Department, Montpellier	33 (7.5)	11 (1.5)	
Rheumatology Department, Toulouse	30 (6.9)	3 (0.4)	
Metabolic disease Department, Nîmes	119 (27.2)	226 (31.6)	
Type of care (*n* = 1075)			<0.01
Consultation	167 (40.7)	431 (64.8)	
Full-time hospitalization	55 (13.4)	130 (19.5)	
Day hospitalization	184 (44.9)	43 (6.5)	
Weekly hospitalization	3 (0.7)	21 (3.2)	
Teleconsultation	1 (0.2)	40 (6.0)	
Chronic pathologies			
Diabetes	-	714 (100)	
Diabetes type (*n* = 714)			
Type 1	-	264 (37.0)	
Type 2	-	450 (63.0)	
Obesity (*n* = 1150)	126 (28.9)	46 (6.4)	<0.01
Active cancer (*n* = 1150)	17 (3.9)	2 (0.3)	<0.01
Chronic inflammatory rheumatism (*n* = 1151)	28 (6.4)	3 (0.4)	<0.01
Chronic inflammatory bowel disease (*n* = 1150)	169 (38.8)	11 (1.5)	<0.01
Chronic renal failure or renal transplantation (*n* = 1150)	53 (12.2)	42 (5.9)	<0.01
Chronic heart failure (*n* = 1150)	19 (4.4)	25 (3.5)	0.46
Others cardiac diseases (*n* = 1150)	15 (3.4)	3 (0.4)	<0.01
Liver transplantation (*n* = 1150)	15 (3.4)	5 (0.7)	<0.01
Chronic respiratory disease (*n* = 1150)	14 (3.2)	6 (0.8)	<0.01
Duration of primary disease, years (*n* = 858)	12.2 (±10.2)	17.4 (±12.9)	<0.01

Data are the mean ± SD, or *n* (%).

**Table 2:** Patient feelings about infectious risk and knowledge about vaccination.

	Person without Diabetes	Person with Diabetes	*p*
Do you think your medical condition is associated with an increased risk of infection?	*n* = 436	*n* = 709	0.15
Yes	220 (50.5)	386 (54.4)	
I don’t know	79 (18.1)	138 (19.5)	
No	137 (31.4)	185 (26.1)	
Do you think you are vulnerable to the flu virus because of your medical condition?	*n* = 434	*n* = 711	0.02
Yes	249 (57.4)	463 (65.1)	
I don’t know	46 (10.6)	72 (10.1)	
No	139 (32.0)	176 (24.7)	
Do you think you are vulnerable to pneumococcal infection because of your medical condition?	*n* = 433	*n* = 705	0.22
Yes	134 (30.9)	234 (33.2)	
I don’t know	170 (39.3)	294 (41.7)	
No	129 (29.8)	177 (25.1)	
Did the COVID-19 pandemic make you aware that your medical condition is a risk factor for infection?	*n* = 424	*n* = 681	<0.01
Yes	241 (56.8)	456 (67.0)	
I don’t know	51 (12.0)	60 (8.8)	
No	132 (31.1)	165 (24.2)	
Do you know which vaccines are recommended for your condition?	*n* = 422	*n* = 673	
Yes	145 (34.4)	223 (33.1)	0.68
If yes, which ones			
Flu vaccine	82 (56.5)	104 (46.6)	0.06
Pneumococcal vaccine	39 (26.9)	23 (10.3)	<0.01
Tdap (tetanus, diphtheria, pertussis) vaccine	6 (4.1)	14 (6.3)	0.38
COVID-19	7 (4.8)	30 (13.4)	<0.01
For vaccines you are not up to date on, will the COVID-19 pandemic cause you to get the pneumococcal vaccine?	*n* = 203	*n* = 369	0.18
Yes	55 (27.1)	120 (32.5)	
For vaccines you are not up to date on, will the COVID-19 pandemic cause you to get a flu vaccination?	*n* = 189	*n* = 211	0.03
Yes	39 (20.6)	27 (12.8)	

Data are *n* (%).

**Table 3:** Reasons for non-vaccination.

	Flu Vaccine			Pneumococcal Vaccine			Flu vs. Pneumococcal Vaccines (Whole Population)
If you didn’t receive the vaccine this year, it’s because	Person without diabetes (*n* = 187)	Person with diabetes (*n* = 215)	*p*	Person without diabetes (*n* = 192)	Person with diabetes (*n* = 357)	*p*	*p*
You don’t trust vaccination (yes)	27 (14.4)	44 (20.5)	0.11	11 (5.7)	11 (3.1)	0.13	<0.0001
You were not aware of the need for this vaccine (yes)	28 (15.0)	11 (5.1)	<0.01	134 (69.8)	270 (75.6)	0.14	<0.0001
You are afraid of potential side effects (yes)	41 (21.9)	56 (26.0)	0.34	13 (6.8)	24 (6.7)	0.98	<0.0001
You feel that this vaccine is not useful because you do not consider yourself at risk of infection	44 (23.5)	45 (20.9)	0.53	19 (9.9)	25 (7.0)	0.23	<0.0001
You are opposed to vaccination in general	13 (7.0)	19 (8.8)	0.49	10 (5.2)	14 (3.9)	0.48	0.02
You don’t find the vaccine effective	15 (8.0)	39 (18.1)	<0.01	0 (0.0)	1 (0.3)	1.00	<0.0001
Other	19 (10.2)	24 (11.2)	0.75	19 (9.9)	29 (8.1)	0.48	0.31
You were unable to obtain a vaccine (out of stock) only for flu vaccine	35 (18.7)	24 (11.2)	0.03	-	-	-	NA

Data are *n* (%).

**Table 4:** Feelings about vaccines and the impact of the COVID-19 pandemic.

	Before COVID-19 Pandemic			During COVID-19 Pandemic		
	Person without diabetes	Person with diabetes	*p*	Person without diabetes	Person with diabetes	*p*
Feelings about vaccine	*n* = 423	*n* = 656	0.21	*n* = 402	*n* = 614	0.97
For	221 (52.2)	358 (54.6)		201 (50.0)	310 (50.5)	
Against	19 (4.5)	46 (7.0)		37 (9.2)	60 (9.8)	
Mixed	140 (33.1)	195 (29.7)		127 (31.6)	192 (31.3)	
No opinion	43 (10.2)	57 (8.7)		37 (9.2)	52 (8.5)	

Data are mean ± SD or *n* (%).

## Data Availability

The data presented in this study are available on request from the corresponding author. The data are not publicly available due to ethical restrictions.
